# Salivary egf concentration in adults with reflux chronic laryngitis before and after treatment: preliminary results

**DOI:** 10.1016/S1808-8694(15)31060-0

**Published:** 2015-10-22

**Authors:** Claudia Alessandra Eckley, Lilia da Silva Rios, Luiz Vicente Rizzo

**Affiliations:** 1 Doctorate in Medicine, FCMSCSP. Professional Voice Fellow, Thomas Jefferson University - Philadelphia. Assistant Professor of the Santa Casa Otorhinolaryngological Department, Sao Paulo.; 2 Master's degree in Biomedicine, Sao Paulo University, Graduate student in the Sao Paulo University Immunology Department.; 3 Full Professor of the Immunology Department of the USP Biomedical Science Institute. Santa Casa Medical School, SP.

**Keywords:** gastroesophageal reflux disease (gerd), epidermal growth factor (egf), chronic laryngitis, saliva

## Summary

**T**he Laryngopharyngeal Reflux (LPR) physiopathology is still unknown. The Epidermal Growth Factor (EGF) is a biologically active salivary protein that aids in the rapid regeneration of the oropharyngeal and upper digestive tract mucosas. Salivary deficiency of this protein in patients with LPR has been demonstrated in previous studies. **Aim:** To compare salivary EGF concentration in patients with LPR before and after treatment. **Materials and Methods:** In this prospective study twelve patients with GERD and moderate LPR were studied. Whole saliva samples were collected before and after treatment and salivary EGF concentration was determined using a commercially available ELISA kit (Quantikine ®). **Results:** There were eleven females and one male among the patients, the mean age was 49 years. The mean pre-treatment salivary EGF concentration was 2,867.6 pg/mL and the mean post treatment EGF concentration was 1,588.5 pg/mL. This difference was statistically significant (p=0.015). **Discussion and Conclusions:** Although salivary EGF concentrations are higher before LPR treatment, the concentration is still much lower than the mean salivary EGF concentration in normal individuals without LPR, which suggests a primary disorder of this defense factor in individuals with LPR.

## INTRODUCTION

Gastro-esophageal reflux disease (GERD) is the most prevalent digestive disorder of modern times, in the last decade it has been implicated in a number of laryngo-pharyngeal disorders^1^, ^2^, ^3^, ^4^, ^5^, ^6^, ^7^, ^8^, ^9^, ^10^, ^11^, ^12^, ^13^, ^14^, ^15^, ^16^, ^17^. This supraesophageal form of the GERD was called Laryngo-Pharyngeal Reflux (LPR) by Koufman et al. in 1994^7^, not aiming at establishing its origin, but rather with the intent of stressing symptoms predominance and the alterations brought about to the laryngopharyngeal segment. Symptoms associated to the reflux are weekly reported by 3 to 6% of the individuals in the general population^13^,^18^,^19^. Notwithstanding, very little is known about the physiopathology of these supraglottic GERD presentations.

It is interesting to notice how a large number of patients with laryngopharyngeal reflux (LPR), even those with more marked laryngeal findings, do not have esophagitis or other signs of GERD in their digestive tract^15^, ^16^, ^17^. Certainly, the gastric and esophageal mucosal protection mechanisms have a decisive role in the capacity these organs have of withstanding mechanical and chemical aggression to which they are daily exposed, and many of these mechanisms are saliva-mediated^15^,^20^, ^21^, ^22^, ^23^, ^24^, ^25^, ^26^, ^27^. Saliva has many organic and inorganic substances that contribute to this protection against physical and chemical attacks, and used for the maintenance of the mucosal lining, not only of the oral mucosa, but also that of the digestive tract^20^, ^21^, ^22^, ^23^, ^24^, ^25^, ^26^. Contradictory clinical findings and recent research reports suggest deficiencies in the defense capacity of this segment in GERD^22^, ^23^, ^24^ patients and, more specifically, in LPR^15^, 27, 28, 29.

One of the factors most responsible for homeostasis of the oral mucosa and the digestive tract is the saliva and its organic content. The Epidermal Growth Factor - EGF, is the salivary protein with the most action on epithelial regeneration after physical and chemical aggression, because of its important capacity in replicating DNA and aiding in the neoangiogenesis of epithelial cells^25^. In a recent study, we proved that there is a significant EGF concentration reduction in saliva in individuals with LPR when compared to normal individuals^27^. Our goal with the present investigation is to check and see if there are salivary EGF concentration alterations in the same individual with GERD and LPR before and after clinical treatment, in order to try and establish if the deficiency of this protection factor is primary (congenital) or secondary (acquired).

## MATERIALS AND METHODS

**Figure 1:**
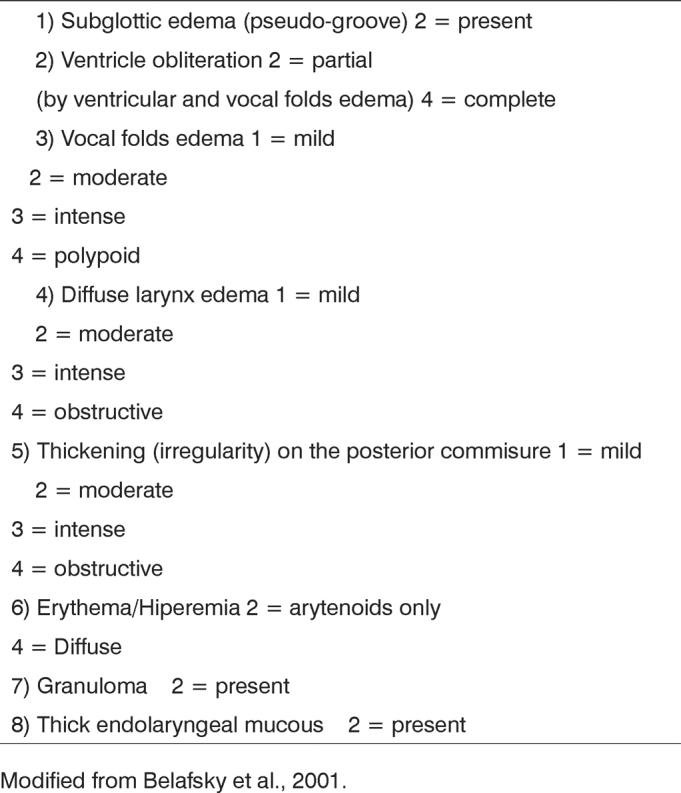
Laryngopharyngeal reflux-caused-inflammatory process intensity scale, based on videolaryngoscopy signs (varies from zero to 28).

Modified from Belafsky et al., 2001.

Our series is made up of 12 individuals, eleven females and one male, with average age of 49 years (ranging between 33 and 72 years), with clinical diagnosis of GERD confirmed by 24 hour two-channel esophageal pH-measurement, screened in an Otorhinolaryngology ward of a tertiary University Hospital. This research project was approved by the Ethics Committee of our Institution (Protocol # CEP 179/04). We only included in our study those patients who had endoscopic diagnosis of GERD, corroborated by 24hour two-channel pH-measures, who agreed to participate in the study after having been explained is goals, procedures used and risks involved. Exclusion criteria encompassed smoking, alcohol intake and exposure to volatile chemical abrasive substances - because all these factors cause inflammation in the respiratory mucosa and may mimic the alterations seen in GERD. Moreover, we also excluded those patients who had used gastric secretion blocking agents, pro-kinetics, anti-acids or hormonal and non-hormonal anti-inflammatory agents in the 14 days prior to their inclusion in the protocol, because these drugs impact the digestive tract mucosa and the gastric secretion. We also excluded patients with larynx and pharynx pre-neoplastic or neoplastic lesions (either present or previously treated). Individuals with intolerance to proton-pump inhibitors did not participate in the study. All participants answered a detailed questionnaire about their general health and GERD-related symptoms, their digestive and otorhinolaryngological manifestations^15^,^16^. They also underwent videolaryngoscopy exam with a 3.5mm Pentax flexible scope, which were recorded in a DVD by using the score established by Belafasky et al. in 2001 (Reflux Finding Score - RFS) (Figure 1)^30^. Nasal-fibro-laryngoscopy was carried out right at the start of the protocol (pre-treatment exam) and after the end of treatment and disease control (post-treatment exam). As disease control we deem to be the resolution of the laryngopharyngeal symptoms and RFS improvement. The standardized treatment for all the patients was the use of a proton inhibiting drug in a full dose, before breakfast and before dinner during 16 weeks. After this period, the patients were reassessed from the standpoint of clinical signs and symptoms of LPR, and a new saliva collection was carried out 7 days after the medication was interrupted.

The participants of this study had two samples of their saliva collected during 15 minutes after a 12 hour fasting period, one pretreatment and another post-treatment (harvested seven days after the drug treatment interruption). The patients were instructed not to use toothpaste on the sample-harvesting day, and all the samples were harvested in the same morning period in order to comply with circadian salivary production fluctuations28. The samples were immediately packed in ice, in order to avoid protein denaturation. To study the EGF salivary content, the total saliva supernatant material was used after being centrifuged for 10 minutes at room temperature at a speed of 5000-7000 revolutions per minute (RPM). This material was then transferred to another Falcon tube with a threaded cap and was stored at −70 degrees Celsius until its analysis. The samples received alpha-numerical identification. In order to determine the salivary EGF concentration we used a commercially available ELISA kit for EGF dosing (Quantikine R) supplied by R&D Systems Inc., EUA. The EGF concentration was determined based on saliva protein concentration. We compared EGF salivary concentrations before and after treatment, as well as correlations with symptoms improvement and laryngeal inflammatory findings.

Results were statistically analyzed and plotted by means of the Wilcoxon test, with a 95% significance level.

## RESULTS

**Figure 2:**
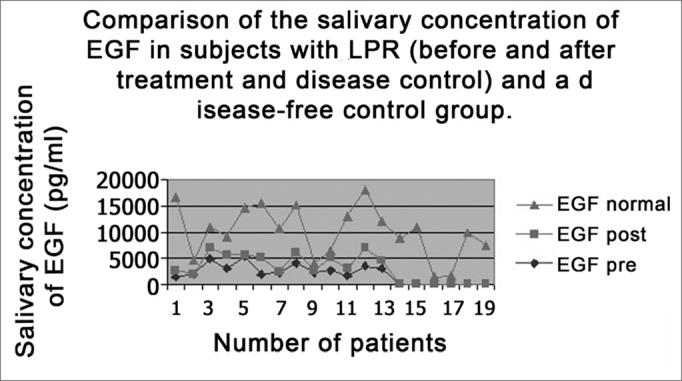
EGF salivary concentration mean values in the two study periods (pre and post treatment) with EGF salivary concentration average in a control population (without reflux).

All subjects in the study had moderate LPR with daily laryngopharyngeal symptoms and an average RFS score of 21.4 points. After treatment, patients remained symptom less in both the laryngopharyngeal and digestive tracts, and the average RFS dropped 13 points (Table 1). Pre-treatment average EGF salivary concentration was of 2,867.6pg/mL and the post treatment and disease control concentration was of 1,588.5pg/mL, and such difference was statistically significant (p=0.015) (Table 1). When we compare the average EGF salivary concentration of this sample with the EGF salivary concentration of a previously established population of normal adults^15^,^27^, we notice that normal individuals presented in average more salivary EGF than individuals with LPR (7,085pg/mL versus 2,867.6pg/mL and 1,588.5pg/mL pre and post treatment, respectively). This difference between individuals without the disease and those with LPR was statistically significant (p=0.02) (Figure 2). There was no difference in data when the only male individual of the sample was excluded from the statistical analysis. Thus, we kept this patient's data in the studied group.

**Table 1:** Demographic and salivary EGF (Epidermal Growth Factor) concentration data of individuals in the study before and after treatment with IBP for 16 weeks.

Patient	gender	age (years)	Initial RFS	Final RFS	PreEGF(pg/mL))	PostEGF (pg/mL)
1	F	36	22	13	1463,35	1071,25
2	M	54	21	14	1874,7	56
3	F	64	22	12	4717,05	2231,15
4	F	72	20	16	3006,65	2661,3
5	F	60	23	12	5337,3	365,75
6	F	33	22	14	1758,5	3316,15
7	F	46	22	12	2147,35	209,75
8	F	45	20	14	3986,3	2072,95
9	F	38	16	9	2243,65	303,7
10	F	45	24	16	2633,4	2370,35
11	F	52	24	12	1729,35	1119,2
12	F	47	21	12	3516,15	3288,2
average		49,33	21.4	13	2867,5	1588,5

## DISCUSSION AND CONCLUSIONS

The supraesophageal forms of GERD represent a somewhat new concept for the scientific community. When of the first descriptions of laryngopharyngitis cases associated to reflux episodes, there was much debate and lack of understanding about diagnostic and treatment modalities, since most assumptions valid for the classic GERD did not seem to apply to its laryngeal manifestation. In this last decade, we have learned to trust the findings from otorhinolaryngologists, by means of videolaryngoscopy, not ruling out the diagnosis of such disease when the patient presented normal or slightly altered esophageal endoscopic exam. PH metrics have also improved, and we now have two measuring channels in order to better assess those cases of physiologic reflux to the digestive tract, but pathologic to the pharynx and larynx^5^,^8^,^11^. National^31^ and Internacional^32^,^33^ consensuses were carried out in order to enhance interdisciplinary communication and better approach the cases of GERD and LPR. Notwithstanding, we are still unable to fully understand the physiopathology of such disease in our modern Western society. There is no correlation between the intensity of endoscopic findings, both in the digestive tract and in the laryngopharyngeal segment, and episodes intensity and frequency when measured by long duration pH-metrics^30^,^33^. Individuals with the same intensity of reflux episodes may have totally distinct mucosal inflammatory alterations. This strengthens the possibility of existing predisposing genetic biomolecular co-factors for this disease, as we have already determined for so many other chronic diseases. Studies carried out before the present one, have established salivary and digestive tract deficiencies of protection factors, such as EGF, in individuals with dyspeptic disease or reflux esophagitis^21^,^22^,^25^,^26^. More recently we have established a significant reduction in EGF salivary concentration in individuals with GERD chronic laryngitis when compared to a group of normal individuals^15^,^16^,^27^.

In the present study we found an EGF salivary concentration significantly higher in individuals during disease activity that dropped after the disease was controlled. This higher EGF salivary concentration during disease activity probably shows an attempt our body make in order to defend itself from the chemical aggression to which it is being subjected. However, this significant difference in salivary concentration of such epithelium regenerator polypeptide between normal individuals and those with LPR suggest the possibility of primary deficiency in salivary production of this important defense factor, further reinforcing the greater susceptibility of some individuals in developing inflammatory alterations on the laryngopharyngeal mucosa after exposure to the chemical aggression caused by the gastroduodenal content reflux.

Both GERD and LPR are known to prevail in females^3^,^6^,^8^,^15^,^16^, and this explains why we have only one man in this series. Notwithstanding, the data of this single patient was kept because it was homogenous with those from the other female participants. The present study reports on the preliminary results of a larger study aiming at better understanding the physiopathological factors related to this atypical form (but not so atypical for otorhinolaryngologists) of Gastro-Esophageal Reflux Disease. If, in fact, this primary deficiency in organic defense mechanisms of the laryngopharyngeal segment is proven, we may foresee much less aggressive and anti-physiologic ways to diagnose and treat Laryngopharyngeal Reflux. Despite our small sample, findings were highly significant, suggesting a strong correlation between EGF salivary concentration deficiency and LPR.
